# *Aspergillus fumigatus*-Related Species in Clinical Practice

**DOI:** 10.3389/fmicb.2016.00683

**Published:** 2016-05-17

**Authors:** Frédéric Lamoth

**Affiliations:** Infectious Diseases Service, Department of Medicine, and Institute of Microbiology, Lausanne University HospitalLausanne, Switzerland

**Keywords:** *Aspergillus* section *Fumigati*, *Aspergillus lentulus*, *Aspergillus udagawae*, *Aspergillus viridinutans*, *Aspergillus felis*, *Neosartorya fischeri*, *Neosartorya pseudofischeri*, *Neosartorya hiratsukae*

## Abstract

*Aspergillus fumigatus* is the main etiologic agent of invasive aspergillosis (IA). Other *Aspergillus* species belonging to the section *Fumigati* (*A. fumigatus* complex) may occasionally be the cause of IA. These strains are often misidentified, as they cannot be distinguished from *A. fumigatus* by conventional morphological analysis and sequencing methods. This lack of recognition may have important consequences as these *A. fumigatus*-related species often display some level of intrinsic resistance to azoles and other antifungal drugs. *A. lentulus*, *A. udagawae, A. viridinutans*, and *A. thermomutatus* (*Neosartorya pseudofischeri*) have been associated with refractory cases of IA. Microbiologists should be able to suspect the presence of these cryptic species behind a putative *A. fumigatus* isolate on the basis of some simple characteristics, such as defect in sporulation and/or unusual antifungal susceptibility profile. However, definitive species identification requires specific sequencing analyses of the beta-tubulin or calmodulin genes, which are not available in most laboratories. Multiplex PCR assays or matrix-assisted laser desorption ionization – time-of-flight mass spectrometry (MALDI-TOF MS) gave promising results for rapid and accurate distinction between *A. fumigatus* and other *Aspergillus* spp. of the section *Fumigati* in clinical practice. Improved diagnostic procedures and antifungal susceptibility testing may be helpful for the early detection and management of these particular IA cases.

## Introduction

*Aspergillus fumigatus* is the most important pathogenic filamentous fungus in humans causing a spectrum of diseases including allergic bronchopulmonary aspergillosis, chronic pulmonary aspergillosis and invasive aspergillosis. Among the growing population of patients with depressed immune defenses, *A. fumigatus* represents one of the major infectious cause of death. Other important pathogenic *Aspergillus* spp. include *A. flavus*, *A. niger*, *A. terreus, A. versicolor*, *A. calidoustus*, and *A. nidulans* (Steinbach et al., 2012). These designations actually represent sections (or complexes) of closely related species (also referred as cryptic species) that cannot be clearly distinguished morphologically. While *A. fumigatus sensu stricto* represents by far the leading human pathogen, some other species belonging to the *A. fumigatus sensu latu* complex (i.e., section *Fumigati*) have been recognized as occasional causes of invasive aspergillosis in 3 to 6% of cases (Balajee et al., 2009b; Alastruey-Izquierdo et al., 2013, 2014; Escribano et al., 2013; **Table 1**). Their actual prevalence may be underestimated because of their lack of recognition by conventional diagnostic approaches. Moreover, the diagnosis of invasive aspergillosis often relies on suggestive radiological findings and/or positive fungal biomarkers (galactomannan, beta-1,3-D-glucan; De Pauw et al., 2008), in the absence of microbiological documentation and the actual contribution of *Aspergillus* spp. other than *A. fumigatus* in this setting is unknown.

**Table 1:** Prevalence of *Aspergillus* spp. of section *Fumigati* other than *Aspergillus fumigatus* in clinical specimens.

Country	*Aspergillus* section *Fumigati* N strains	Strains other than *A. fumigatus* N strains (%)	Species (N)	Reference
United States	147	8 (5.4%)	*A. lentulus* (4)	Balajee et al., 2009b
			*A. udagawae* (3)	
			*A. thermomutatus*^1^ (1)	
Spain	362	19 (5.2%)	*A. lentulus* (9)	Escribano et al., 2013
			*A. novofumigatus* (6)	
			*A. udagawae* (2)	
			*A. viridinutans* (2)	
Spain	162	6 (3.7%)	*A. lentulus* (3)	Alastruey-Izquierdo et al., 2013
			*A. viridinutans* (1)	
			*A. thermomutatus*^1^ (1)	
			*A. fumigatiaffinis* (1)	
Portugal	29	1 (3.4%)	*A. lentulus* (1)	Sabino et al., 2014


*^1^Teleomorph: *Neosartorya pseudofischeri.**

Apart from *A. fumigatus*, the species of the section *Fumigati* that are most frequently recovered in clinical specimens and associated with invasive fungal diseases are *A. lentulus*, *A. udagawae*, *A. viridinutans*, *A. thermomutatus* (*Neosartorya pseudofischeri*), *A. novofumigatus*, and *A. hiratsukae* (Balajee et al., 2006, 2009b; Alcazar-Fuoli et al., 2008; Alastruey-Izquierdo et al., 2013; Escribano et al., 2013). Their limited pathogenic role compared to *A. fumigatus* may be explained by a lower thermotolerance and different profiles of secondary metabolites with decreased production of mycotoxins, such as gliotoxin (Frisvad and Larsen, 2015; Tamiya et al., 2015). However, while resistance to triazoles is rare among *A. fumigatus sensu stricto*, these sibling or cryptic species commonly exhibit decreased susceptibility to azoles and other antifungal agents (Alcazar-Fuoli et al., 2008; Escribano et al., 2013; Alastruey-Izquierdo et al., 2014). A recent multicentre prospective survey reported a rate of azole resistance of 3.2% among *A. fumigatus* isolates (van der Linden et al., 2015). Among these resistant strains, 78% were *A. fumigatus sensu stricto* harboring mutations of the *Cyp51A* gene, while the remaining 22% were actually sibling species (*A. lentulus*, *A. thermomutatus*, and *A. udagawae*). Microbiologists and clinicians thus must be aware of the existence of these sibling species that may be the cause of refractory cases of invasive aspergillosis. The aim of this review is to provide an overview of the characteristics of the most clinically relevant *Aspergillus* spp. of section *Fumigati* (other than *A. fumigatus*).

## Taxonomy and Diversity of *Aspergillus* spp. in Section *Fumigati*

The genus *Aspergillus* is divided in subgenera and sections (Peterson, 2008). *A. fumigatus* belongs to the section *Fumigati*, in which 63 species have now been described, although some of them are doubtful because of possible synonymy with other species (Samson et al., 2007; Frisvad and Larsen, 2015). Species delimitation within section *Fumigati* relies on a polyphasic approach including phenotypic characteristics, rate of growth at variable temperatures, extrolite patterns, and genotyping characterization by random amplification of polymorphic DNA-polymerase chain reaction (RAPD-PCR) and multilocus sequence typing (Hong et al., 2005; Samson et al., 2007). Based on this approach, Yaguchi et al. (2007) have proposed a subdivision of the section *Fumigati* into five clades: (I) *A. fumigatus*, (II) species including *A. lentulus* and *A. fumisynnematus*, (III) species including *A. fumigatiaffinis* and *A. novofumigatus*, (IV) *A. viridinutans*, *A. udagawae*, and other atypical strains, and (V) species including *A. hiratsukae*, *A. brevipes*, *A. duricaulis*, and *A. unilateralis*.

Teleomorphic homothallic species producing ascomata (i.e., sexual stage of reproduction) were initially classified under the genus name *Neosartorya*. However, many species thought to be strictly anamorphic further demonstrated the ability to reproduce sexually with an opposite mating type (e.g., *A. fumigatus*, and *A. lentulus*; O’Gorman et al., 2009; Swilaiman et al., 2013). From 2012, taxonomists recommend to apply a single-name nomenclature keeping the name *Aspergillus* for all species of this genus (Samson et al., 2014). This system will be used in this review with reference to the old teleomorphic name in brackets when applicable.

## Identification of *Aspergillus* spp. of Section *Fumigati*

Morphological examination of macroscopic and microscopic features remains the standard method for identification of filamentous fungi. While phenotypic characteristics allow distinguishing *Aspergillus* spp. at the section level, accurate species identification is usually not possible. Compared to *A. fumigatus sensu stricto*, other *Aspergillus* spp. of the section *Fumigati* may exhibit distinct morphological features, such as loss of pigmentation, poor sporulation, presence of ascomata, or variable growth at different temperature (Balajee et al., 2005a; Hong et al., 2005). These distinctive features, summarized in **Table 2**, are non-specific and variable, according to the growth media and conditions. Molecular methods are increasingly used as an adjunctive diagnostic tool in medical mycology. However, sequencing methods usually targeting fungal ribosomal DNA [internal transcribed spacer region (ITS), 18S rDNA, 26S/28S rDNA] are reliable for distinction at the section level, but not at the species level for *Aspergillus* spp. (Balajee et al., 2007). Phylogenetic studies integrate data of partial DNA sequences of various genes, such as beta-tubulin (*benA*), calmodulin, actin and hydrophobin (*rodA*), to distinguish the different species within the section *Fumigati* (Hong et al., 2005; Samson et al., 2007; Yaguchi et al., 2007). This approach is fastidious and not convenient for rapid identification in clinical practice. Currently, experts recommend comparative sequence analyses of the ribosomal ITS region (ITS1 and ITS2 flanking regions of the 5.8S rDNA) for intersection identification of *Aspergillus* spp. and of the beta-tubulin or calmodulin genes for intrasection identification at the species level (Balajee et al., 2007, 2009a; Samson et al., 2014). Multiplex PCR assays targeting microsatellite markers or specific gene sequences (ITS2 region, *benA*, *rodA*) have been developed for rapid detection and discrimination of *Aspergillus* species within the section *Fumigati* in clinical samples (Serrano et al., 2011; Araujo et al., 2012; Fernandez-Molina et al., 2014). Other techniques have also been described, such as PCR-restriction fragment length polymorphism (PCR-RFLP) of the *benA* gene (Staab et al., 2009) and microsphere-based Luminex assay (Etienne et al., 2009).

**Table 2:** Phenotypic characteristics of the most clinically relevant *Aspergillus* spp. of section *Fumigati.*

Species	Growth				Macroscopic (days 3–7)^3^	Microscopic	Antifungal susceptibility^4^		
				
	10	37	48	Max T			AMB	TAZ	ECH
*A. fumigatus*	-	++	+	>55°C	Velvety, Green–blue	Vesicles: pyriform (22 μm diameter) Conidial head: columnar	++	++	++
*A. lentulus*	+	++	-	45°C	Floccose, whitish, pale green-blue (poor sporulation)	Vesicles: globose, smaller (15 μm) Conidial head: shorter	+/-	+/-^5^	+/-^6^
*A. udagawae*	+	++	-	42°C	Floccose, whitish, pale green–blue (poor sporulation) Pink–purple exudate	Vesicles: subglobose to globose, smaller (15 μm)	+	+^5^	+^6^
*A. viridinutans*/*felis*	-	+(+)^1^	-	40–45°C^2^	Floccose, whitish to Niagara green	Vesicles: subglobose, smaller (8–15 μm) Conidial head: “nodding”	+/++	+/-^5^	+^6^
*A. fischeri*/*thermomutatus*^1^	NA	++	(+)	48°C	Velvety, whitish (poor sporulation)	Few ascomata (cleistothecia) at 37°C, abundant at 25°C	++	+ / - 5	+^6^


*Phenotypic characteristics are expressed in comparison to *A. fumigatus* reference wild-type strain (Af293). Max T, maximal temperature at which growth may be observed; AMB, amphotericin B; TAZ, triazoles (voriconazole, posaconazole, and itraconazole), ECH: echinocandins (caspofungin, micafungin, and anidulafungin); NA, no data available ^1^Teleomorph: *Neosartorya fischeri/pseudofischeri.*^2^*Aspergillus felis* exhibits better growth at 37°C and is able to growth at 45°C, while *A. viridinutans* cannot. ^3^Aspect on conventional fungal culture media, such as Czapek, Sabouraud dextrose agar or potato dextrose agar. ^4^Antifungal susceptibility is expressed according to minimal inhibitory concentrations (MIC) for fungicidal drugs (amphotericin B, triazoles) and minimal effective concentrations (MEC) for fungistatic drugs (echinocandins), based on data reported from the literature. Grading was assessed as follows: ++ (MIC ≤ 1 μg/ml or MEC ≤ 0.25 μg/ml); + (MIC 1–4 μg/ml or MEC 0.25–2 μg/ml), - (MIC > 4 μg/ml or MEC > 2 μg/ml). These data are indicative and may display important variations according to each individual isolate. ^5^Better *in vitro* activity of posaconazole compared to voriconazole and itraconazole. ^6^Better *in vitro* activity of micafungin and anidulafungin compared to caspofungin.*

Matrix-assisted laser desorption ionization – time-of-flight mass spectrometry (MALDI-TOF MS) also gave promising results for the distinction between *A. fumigatus* and *A. lentulus* in clinical isolates (Verwer et al., 2014). This approach demonstrated accuracy for identification of *Aspergillus* spp. at the species level (Sanguinetti and Posteraro, 2014). Mass spectra of *A. lentulus* and other cryptic *Aspergillus* spp. have been included in different commercial or in-house libraries. However, protein extraction procedures for filamentous fungi are not standardized and further analyses of mass spectra of these fungal species, performed under the same experimental conditions, are warranted in order to obtain a complete reference database.

## Clinically Relevant *Aspergillus* spp. of Section *Fumigati* (Other than *A. fumigatus*)

Various cases of fungal infections involving *Aspergillus* spp. of section *Fumigati* other than *A. fumigatus* have been reported in the medical literature (**Table 3**). Clinical presentation is often similar to that of other invasive aspergillosis and positive fungal biomarkers (galactomannan, beta-1,3-D-glucan) may also be observed. *A. lentulus* is the most frequent species recovered in clinical specimens and mixed infections with concomitant *A. fumigatus* are also reported. Invasive fungal infections due to *A. lentulus* or other *A. fumigatus*-related species are associated with a particularly high mortality rate (about 60%). In addition, these cryptic species have also been associated with allergic fungal diseases and chronic colonization in patients with cystic fibrosis (Shivaprakash et al., 2009; Symoens et al., 2010). Some microbiological and clinical features of the most clinically important species will be discussed in this chapter.

**Table 3:** Case reports of invasive aspergillosis attributed to *Aspergillus* spp. of section *Fumigati* other than *A. fumigatus.*

Species (N cases)	Host risk factors	Type of infection	Outcome	Reference
*A. lentulus* (6)	SOT (4)	IPA	Success (1)	Zbinden et al., 2012; Escribano et al., 2013; Gurcan et al., 2013; Bastos et al., 2015; Yoshida et al., 2015
	COPD/steroid (2)		Failure (4)	
			NR (1)	
*A. udagawae* (6)	CGD (3)	IPA (5)^3^	Success (3)	Vinh et al., 2009b; Posteraro et al., 2011; Gyotoku et al., 2012
	HM (1)	Keratitis (1)	Failure (3)	
	None (2)			
*A. thermomutatus* (10)^1^	HM (3)	IPA (1)	Success (5)	Gerber et al., 1973; Coriglione et al., 1990; Summerbell et al., 1992; Padhye et al., 1994; Matsumoto et al., 2002; Jarv et al., 2004; Balajee et al., 2005b; Ghebremedhin et al., 2009; Toskova et al., 2013; Khare et al., 2014
	Peritoneal dialysis (2)	Disseminated IA (2)	Failure (4)	
	Cystic fibrosis (1)	Catheter-related peritonitis (3)	NR (1)	
	None (4)	Soft tissue infections (2)		
		Endocarditis (1)		
		Keratitis (1)		
*A. fischeri* (3)	HM (2)	IPA (1)	Success (1)	Lonial et al., 1997; Chim et al., 1998; Gori et al., 1998
	SOT (1)	Disseminated IA (2)	Failure (2)	
*A. viridinutans*/*A. felis* (5)	SOT (1)	IPA (4)^3^	Success (2)	Katz et al., 2005; Vinh et al., 2009a; Coelho et al., 2011; Shigeyasu et al., 2012
	CGD (1)	Keratitis (1)	Failure (2)	
	Job syndrom (1)		NR (1)	
	Rheumatoid arthritis (1)			
	None (1)			
*A. hiratsukae* (3)	Peritoneal dialysis (2)	Cerebral aspergillosis (1)	Success (1)	Guarro et al., 2002; Predari et al., 2007; Koutroutsos et al., 2010
	None (1)	Catheter-related peritonitis (2)	Failure (2)	
Mixed^2^ (8)	HM (3)	IPA (8)	Success (1)	Alhambra et al., 2008; Montenegro et al., 2009; Escribano et al., 2013; Pelaez et al., 2013
	Solid tumor (3)		Failure (7)	
	COPD (1)			


*^1^Teleomorph: *N. pseudofischeri.*^2^*Aspergillus lentulus*/*A. fumigatus* (4), *A. lentulus*/*A. udagawae*/*A. fumigatus* (1), *A. lentulus*/*A. novofumigatus*/*A. fumigatus* (1), *A. udagawae*/*A. fumigatus* (1), *A. felis*/*A. novofumigatus*/*A. calidoustus* (1). ^3^Contiguous extension from lung to adjacent structures frequently reported. SOT, solid-organ transplantation; COPD, chronic obstructive pulmonary disease; CGD, chronic granulomatous disease; HM, hematologic malignancy; IPA, invasive pulmonary aspergillosis; NR, not reported.*

## Aspergillus lentulus

*Aspergillus lentulus* was first identified in 2004. While retrospectively screening for itraconazole resistance in a collection of 128 *A. fumigatus* isolates obtained from hematopoietic stem cell transplant recipients between 1991 and 2000 in Seattle, Balajee et al. (2004) described an *A. fumigatus* variant exhibiting poor sporulation and decreased susceptibility to azoles, amphotericin B and echinocandins. These atypical isolates were found to be the cause of invasive fungal infections in 4 (5%) of these patients. On the basis of phylogenetic analyses, these isolates were assigned to a new species within the section *Fumigati* under the name of *A. lentulus* (Balajee et al., 2005a). Phenotypic characteristics of *A. lentulus* compared to *A. fumigatus* are poor sporulation, smaller conidial heads and inability to grow at temperature above 48°C. Mass spectrometry methods (MALDI-TOF, SELDI-TOF) showed promising results for rapid discrimination between *A. lentulus* and *A. fumigatus* (Pinel et al., 2011; Verwer et al., 2014).

*Aspergillus lentulus* seems to represent only a small proportion (<3%) of all *Aspergillus* spp. of the section *Fumigati* isolated in clinical specimens (Balajee et al., 2009b; Alastruey-Izquierdo et al., 2013; Escribano et al., 2013). However, it may account for a substantial proportion (10–30%) of azole-resistant isolates that are morphologically classified as *A. fumigatus* (Mortensen et al., 2010; Kidd et al., 2015; van der Linden et al., 2015). Clinical reports of *A. lentulus* infections mainly consist of invasive pulmonary aspergillosis in patients with hematologic malignancies or solid-organ transplant recipients (Balajee et al., 2004, 2009b; Zbinden et al., 2012; Escribano et al., 2013; Gurcan et al., 2013; Bastos et al., 2015; Yoshida et al., 2015). Interestingly, *A. lentulus* is often found in association with *A. fumigatus* in mixed infections with poor outcomes (Alhambra et al., 2008; Montenegro et al., 2009; Escribano et al., 2013). *A. lentulus* was pathogenic in murine models of invasive aspergillosis, but was associated with a decreased mortality and a delayed course of infection compared to *A. fumigatus* (Mellado et al., 2011). *A. lentulus* was also pathogenic in larvae of *Galleria mellonella* who did not respond to voriconazole therapy (Alcazar-Fuoli et al., 2015).

Decreased susceptibility to all three antifungal classes (azoles, polyenes and echinocandins) are reported in most clinical isolates (Alcazar-Fuoli et al., 2008; Alastruey-Izquierdo et al., 2013, 2014; Escribano et al., 2013). The level of resistance of *A. lentulus* to triazoles is variable, with higher MICs observed for voriconazole and itraconazole compared to posaconazole. The new triazole isavuconazole displays good *in vitro* activity against *A. lentulus* (Datta et al., 2013). Intrinsic azole resistance in *A. lentulus* is dependant of the *Cyp51A* gene (*AlCyp51A*), which shares 92% homology with its *A. fumigatus* ortholog (*AfCyp51A*). Substitution of *AfCyp51A* by *AlCyp51A* induced the same phenotype of azole resistance in *A. fumigatus* (Mellado et al., 2011). Molecular dynamics modeling suggested that some differences in the BC-loop between AfCyp51A and AlCyp51A may affect the closed form of the protein upon voriconazole binding (Alcazar-Fuoli et al., 2011). Among echinocandins, decreased susceptibility of *A. lentulus* is mainly observed for caspofungin (Alcazar-Fuoli et al., 2008; Alastruey-Izquierdo et al., 2014). However, there was no polymorphisms in the known hot spot regions of the *A. lentulus fks* gene (encoding for the β-1,3-glucan synthase target of echinocandins), which shares 98% homology with *A. fumigatus fks* (Staab et al., 2010).

## *Aspergillus* (*Neosartorya*) *udagawae*

*Aspergillus udagawae* was first isolated in 1995 from the soil of a sugar cane plantation in Brazil (Horie et al., 1995). Morphological features that may help distinguishing *A. udagawae* from *A. fumigatus* are the decreased spore production and the secretion of a pink-purple pigment on agar plates. *A. udagawae* also exhibits a different range of thermotolerance, being able to grow at 10°C, but not at ≥42°C, with an optimal growth at 30–35°C (Sugui et al., 2010). Decreased rates of germination and increased susceptibility to neutrophils and hydrogen peroxide may explain the lower virulence of *A. udagawae* compared to *A. fumigatus* in murine models (Sugui et al., 2010; Gyotoku et al., 2012).

Molecular analyses suggest that *A. udagawae* may account for a small proportion of the *Aspergillus* spp. of section *Fumigati* isolated from clinical specimens (Balajee et al., 2006; Vinh et al., 2009b; Escribano et al., 2013). Few clinical cases have been reported in the literature. *A. udagawae* may cause a chronic form of pulmonary aspergillosis with a propensity for contiguous spread to adjacent structures, which may be refractory to antifungal therapy (Vinh et al., 2009b). Cases of endobronchial infection and post-traumatic keratitis have also been reported in immunocompetent patients (Posteraro et al., 2011; Gyotoku et al., 2012). *A. udagawae* isolates usually exhibit higher MIC (1 to 3 dilutions) to amphotericin B and voriconazole compared to *A. fumigatus sensu stricto* (Balajee et al., 2006; Vinh et al., 2009b). The new triazole isavuconazole demonstrated increased activity (MIC ranging 0.031–0.25 μg/ml; Datta et al., 2013).

## Aspergillus viridinutans/felis

*Aspergillus viridinutans* was first isolated from a rabbit dung in Australia and subsequently documented in some human clinical isolates (Alcazar-Fuoli et al., 2008; Vinh et al., 2009a; Coelho et al., 2011; Escribano et al., 2013; Pelaez et al., 2013). Recently, some specimens previously identified as *A. viridinutans* have been reassigned to the novel closely related species *A. pseudoviridinutans*, *A. felis*, *A. pseudofelis*, and *A. parafelis* (Katz et al., 2005; Barrs et al., 2013; Alvarez-Perez et al., 2014; Sugui et al., 2014). These species sporulate weakly and produce a typical “nodding” conidial head (Barrs et al., 2013). Contrarily to *A. viridinutans*, *A. felis* is able to grow at 45°C. *A. felis* is a common cause of sino-orbital aspergillosis in cats (Barrs and Talbot, 2014). Similar to *A. udagawae*, *A. viridinutans*, and *A. felis* cause human pulmonary aspergillosis characterized by a chronic evolution and spread across contiguous anatomic planes (Vinh et al., 2009a; Coelho et al., 2011). A case of fungal keratitis was also described in human (Shigeyasu et al., 2012). Most clinical isolates showed high MICs for voriconazole and itraconazole (usually ≥4 μg/ml), but lower values for posaconazole and amphotericin B (Alcazar-Fuoli et al., 2008; Vinh et al., 2009a; Coelho et al., 2011; Shigeyasu et al., 2012; Escribano et al., 2013; Pelaez et al., 2013).

## *Aspergillus* (*Neosartorya*) *fischeri*/*Aspergillus thermomutatus* (*Neosartorya pseudofischeri)*

*Aspergillus fischeri* (teleomorph: *N. fischeri*) is a ubiquitous fungus producing thermotolerant ascospores, which is responsible of food spoilage of fruit juices and other heat-processed fruit products. *A. thermomutatus* (teleomorph: *N. pseudofischeri*) can only be differentiate from *A. fischeri* by electron microscopic analysis of the ascospores (Peterson, 1992). These homothallic fungi produce whitish fast-growing colonies with few sporulation at 37°C and can tolerate higher temperatures (48°C). Ascomata of the sexual form are usually abundant, but may be scarce or appear at later stages, especially at 37°C.

*A. fischeri* and *A. thermomutatus* have been identified as the cause of human mycoses in only few case reports with a broad spectrum of disease’s presentation including extrapulmonary manifestations in most cases, such as endocarditis, osteomyelitis, peritonitis, gastro-intestinal tract infection, keratitis, and disseminated infections (Gerber et al., 1973; Coriglione et al., 1990; Summerbell et al., 1992; Padhye et al., 1994; Lonial et al., 1997; Chim et al., 1998; Gori et al., 1998; Matsumoto et al., 2002; Jarv et al., 2004; Balajee et al., 2005b; Ghebremedhin et al., 2009; Toskova et al., 2013; Khare et al., 2014). Susceptibility testing of these isolates usually shows higher MICs for triazoles compared to *A. fumigatus*, but similar values for amphotericin B and echinocandins (Balajee et al., 2005b; Alcazar-Fuoli et al., 2008).

## Other Pathogenic *Aspergillus* spp. in Section *Fumigati*

*Aspergillus* (*Neosartorya*) *hiratsukae* has been identified in some clinical specimens (Alcazar-Fuoli et al., 2008) and has been associated with rare and essentially extrapulmonary fungal diseases, such as cerebral aspergillosis and peritonitis in patients under peritoneal dialysis (Guarro et al., 2002; Predari et al., 2007; Koutroutsos et al., 2010). *A. novofumigatus*, *A. fumigatiaffinis*, and *A. fumisynnematus* have also been isolated in clinical samples, but their pathogenic role remained unclear (Alcazar-Fuoli et al., 2008; Escribano et al., 2013; Pelaez et al., 2013).

## Conclusion

Recent advances in phylogenetic analyses and molecular methods have revealed the important diversity of *Aspergillus* species within the section *Fumigati*. Some clinical isolates may actually be misidentified as *A. fumigatus* by conventional diagnostic methods, which may result in inappropriate antifungal therapy because of the decreased susceptibility of these cryptic species to many antifungal agents. Azole resistance among *A. fumigatus* isolates is an emerging problem, which has been highlighted by multiple recent reports throughout the world, but its prevalence is still low. Actually, in case of refractory invasive aspergillosis, clinicians should suspect that a sibling species of *A. fumigatus* may be the causal agent. Slow or poor sporulation is usually the first hint that should alert the microbiologist. Sequencing of the ITS region, followed by targeted sequencing of the beta-tubulin or calmodulin genes seems to date the most appropriate method for reliable species identification. However, these procedures are not available in most institutions. MALDI-TOF MS may be a very convenient alternative for the rapid detection of *A. lentulus* and other *A. fumigatus*-related species, but further investigations for standardized sample treatment procedures of filamentous fungi and spectra characterization are required. Antifungal susceptibility testing may be useful because of the unpredictable susceptibility profile of these species. However, results should be interpreted with caution because of the lack of clinical breakpoints and the absence of data correlating MICs with clinical outcomes. Recommendations for the antifungal management of such cases cannot be clearly defined on the basis of limited published data consisting in case reports of refractory invasive aspergillosis with often fatal outcomes despite the use of multiple antifungal agents. Decreased susceptibility to azoles is common and higher MICs to other antifungal drugs (amphotericin B, caspofungin) may also be observed, especially for *A. lentulus*. Among triazoles, posaconazole displays better *in vitro* antifungal activity compared to voriconazole and itraconazole, but is not yet approved as first-line therapy of invasive aspergillosis. In addition, the new extended-spectrum isavuconazole, which has recently been approved for the treatment of invasive aspergillosis (Miceli and Kauffman, 2015), may become an interesting alternative as both *A. lentulus* and *A. udagawae* displayed low MICs comparable to that of *A. fumigatus* (Datta et al., 2013). Combination therapies may be considered in refractory cases, although data are lacking to demonstrate a benefit in this setting.

## Author Contributions

FL: Design and redaction of the manuscript.

## Conflict of Interest Statement

The author declares that the research was conducted in the absence of any commercial or financial relationships that could be construed as a potential conflict of interest.
